# Prostate Cancer-Targeting Liposome Loaded with Zinc Ion-Coordinated Photosensitizer for Enhanced Chemo-Photodynamic Therapy

**DOI:** 10.3390/pharmaceutics17040448

**Published:** 2025-03-31

**Authors:** Li Gao, Zhisheng Tang, Dongming Xiao, Xu Chen, Yizhun Zhu

**Affiliations:** 1 State Key Laboratory of Quality Research in Chinese Medicine and School of Pharmacy, Macau University of Science and Technology, Macau 999078, China; 2 College of Pharmacy, Guilin Medical University, Guilin 541004, China; linsdom520@163.com; 3 The Second Affiliated Hospital, Guilin Medical University, Guilin 541199, China

**Keywords:** prostate cancer, zinc ion, photodynamic therapy, tumor targeting, photochemical internalization

## Abstract

**Background:** Prostate cancer (PCa) is the second most prevalent cancer in males globally, impacting one out of every six males. However, the therapeutic effect of chemotherapy on PCa is restricted. **Methods:** To address this, we developed a tumor-targeted multifunctional liposomal platform (PTX-PS/Zn@Lip-Apt) for zinc-enhanced chemo-photodynamic therapy of PCa. Co-delivery of PTX and an aggregation-induced emission photosensitizer (TPEDPD) enables combined chemotherapy and photody-namic therapy. Zinc ions were loaded into liposomes to improve the chemosensitivity of PCa to chemodrugs. Then, the AS1411 aptamer was further modified onto the sur-face of the liposome to enhance its tumor targeting ability. Moreover, to improve the cellular uptake efficiency of the nanoparticles, the photochemical internalization (PCI) strategy was also employed. **Results**: In vitro experiments indicated that aptamer conjugation and PCI application enhanced the cellular uptake and cytotoxicity of PTX/PS-Zn@Lip-Apt. The zinc ion enhanced cytotoxicity could also be found. In vivo experiments demonstrated the good antitumor effect and biosafety of PTX/PS-Zn@Lip-Apt. **Conclusions**: Our findings provide an important basis for innovatively applying zinc-enhanced combined chemo-photodynamic therapy in prostate cancer.

## 1. Introduction

Prostate cancer (PCa) is the second most common cancer in men worldwide [1,2]. As almost all prostate cancers begin in an androgen-dependent way, androgen deprivation therapy is usually administered for prostate cancer. However, some cancerous cells are still able to survive during this treatment and evolve to an androgen-independent prostate cancer [3]. In these cases, chemotherapy, such as docetaxel, will be adopted, but the therapeutic effect is restricted, and fatal cases are usually unavoidable [4,5]. Additionally, because the structure of prostate cancer is close to normal tissue, radiotherapy and surgery may cause a certain degree of damage to normal tissues [6]. Therefore, many novel treatment modalities of prostate cancer have been explored in recent years, including photothermal therapy (PTT), chemodynamic therapy (CDT), and photodynamic therapy (PDT), etc.

PDT is a promising phototherapy strategy and has attracted more and more interest from researchers due to its precise stereospecificity, mild side effects (primarily cutaneous photosensitivity reactions), and minimal invasiveness. [7,8,9,10]. Photosensitizer (PS), which is a key component in PDT, can absorb light energy and effectively convert it into toxic reactive oxygen species (ROS), such as singlet oxygen (^1^O_2_), superoxide anion (O_2_^•−^), and hydroxyl radical (OH). The resulting ROS can damage the organelles of cancer cells and further induce programmed cell death [11]. However, conventional PSs, including boron dipyrromethene, Rose Bengal, methylene blue, cyanine structures, and porphyrin derivatives generally suffer from the intrinsic aggregation-caused quenching (ACQ) effect in aqueous media, resulting in reduced ROS generation and compromised PDT performance [12,13,14]. In recent years, fortunately, the emergence of PSs with aggregation-induced emission (AIE) characteristics has solved this dilemma and triggered the state-of-the-art development of PDT [14,15,16]. Different from traditional fluorophores, the emission of AIE luminogens (AIEgens) is significantly intensified as aggregates owing to a widely accepted AIE emission mechanism, which is the restriction of intramolecular motion (RIM) [17,18,19]. More importantly, instead of being reduced, it has been demonstrated that the ROS generation of AIE PSs is strengthened in the aggregated state [20,21].

Zinc plays an essential role in maintaining structural integrity and catalytic activity of various enzymes, while also contributing to gene stability and expression [22,23]. The prostate is the organ in the human body with the highest level of Zn [24]. In prostate cancer, the reduction in intracellular Zn (II) concentration is a hallmark of malignant transformation and plays a critical role in tumor progression. It has been reported that abnormal concentrations of zinc ions in serum and tissues are associated with tumor occurrence and progression, and a markedly decreased zinc concentration has been observed in patients with prostate carcinoma [25,26]. The concentration of zinc has been shown to decline by 60–70% in the blood serum of patients with prostate cancer, and to decrease further as cancer progresses [25,27]. In the clinic, cancer cells maintaining a higher level of intracellular zinc have been demonstrated to have remarkably slower growth rates [28,29,30]. Some studies have reported that zinc ions could inhibit tumor growth by inhibiting the activity of enzymes in glycolysis including lactate dehydrogenase A (LDHA) and hexokinase II (HK2), disrupting redox metabolism, and activating the cGAS-STING pathway [31,32]. Additionally, several groups have shown that chemosensitivity to some chemotherapeutic drugs can be enhanced when administered simultaneously with zinc ions for prostate cancer cells [24,33]. However, the effect of combined treatment of PDT and chemotherapy with the help of zinc for prostate cancer remains largely unexplored.

Liposomes have become one of the most extensively used nanoparticles (NPs) for the delivery of various anti-cancer agents due to their promising features, including low toxicity, high biocompatibility, high stability, lack of immunogenicity, and high drug loading efficiency [34,35]. Several liposomal formulation-based drugs for cancer treatment have been approved by the FDA, such as Doxil^®^, Onivyde^®^, and Vyxeos^®^ [36]. All these approved liposome drugs reach tumor sites by a passive targeting method called the “enhanced permeability and retention” (EPR) effect [36]. However, passive targeting is not an effective strategy for the delivery of anti-cancer cargos in hypovascular tissues, such as liver, pancreas, and prostate tumors [37,38]. Therefore, how to endow liposomal formulations with active targeting ability and enhance cellular uptake of the liposome or the released drug from the liposome are still the main challenges.

**Figure 1:**
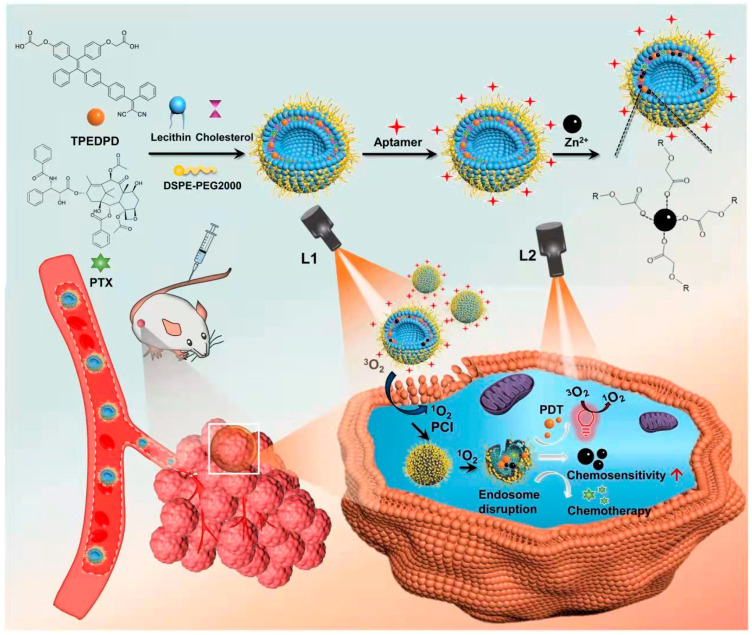
Preparation of PTX/PS-Zn@Lip-Apt. When PTX/PS-Zn@Lip-Apt NPs were accumulated in tumor tissues, the first light irradiation (L1) was utilized to facilitate cellular uptake by “PCI”. Then, the second light irradiation (L2) was used for sufficient ROS production.

In this study, as shown in Figure 1, we co-encapsulated paclitaxel (PTX) and AIE PSs into liposomes and further loaded them with Zn^2+^ by coordination between zinc ions and the carboxyl group of AIE PSs. Then, to enable the therapeutic nanoagent to actively target the prostate tumor and enhance its cellular uptake efficiency, first, we modified the exterior surface of the liposome with AS1411 aptamer (Apt), which can specifically bind to nucleolin (NCL), a protein overexpressed on the plasma membrane of the prostate cancer. Secondly, a photochemical internalization (PCI) strategy was further used. PCI was first developed at the Norwegian Radium Hospital as an efficient strategy for light-enhanced site-specific drug/gene release [39,40]. After being systemically administered, the liposomal nanoagent (named PTX-PS/Zn@Lip-Apt) can actively target the prostate cancer site owning to the existence of Apt. Then, a short-time and insufficient light irradiation is performed to produce a small amount of ROS from PS, which cannot kill cancer cells directly, but can induce lipid peroxidation and improve cytomembrane permeability, resulting in enhanced intracellular uptake of PTX-PS/Zn@Lip-Apt. After internalization, a second light with a stronger power is irradiated to induce PDT and drug release of PTX and Zn^2+^. PTX serves as a chemotherapeutic drug, and the released Zn^2+^ can enhance the combinational treatment effect of PDT and chemotherapy.

## 2. Materials and Methods

### 2.1. Synthesis of Compound **2**

Compound **1** (686 mg, 1.2 mmol), malononitrile (265 mg, 4 mmol), was added to 50 mL dichloromethane. Titanium tetrachloride (0.48 mL, 4.2 mmol) was slowly added to the mixture at 0 °C. After the reaction mixture was stirred for 30 min, pyridine (0.36 mL, 4.2 mmol) was injected and stirred for another 30 min. The reaction mixture was then heated at 50 °C for 6 h. After the mixture was cooled down to room temperature, the reaction was quenched by water (50 mL), and the mixture was extracted with dichloromethane (50 mL × 3). The collected organic layer was washed with brine and dried over Na_2_SO_4_. The solvent was evaporated under reduced pressure followed by purification on silica gel column chromatography with DCM/hexane (1/3) as the eluent to afford compound **2** as a red solid (551.2 mg), resulting in a yield of 74%. ^1^H NMR (400 MHz, CDCl_3_, 298 K) (TMS, ppm): 7.69–7.63 (d, 2H), 7.59 (t, *J* = 6.1 Hz, 1H), 7.47 (dd, *J* = 12.7, 7.9 Hz, 6H), 7.40 (d, *J* = 8.7 Hz, 1H), 7.17–7.03 (m, 7H), 6.97 (dd, *J* = 11.9, 8.9 Hz, 4H), 6.65 (dd, *J* = 8.0, 6.2 Hz, 4H), 3.75 (s, 6H). ^13^C NMR (100 MHz, CDCl_3_, 298 K) (ppm): 174.52, 158.30, 158.21, 136.17, 132.65, 132.14, 131.45, 131.17, 130.53, 128.88, 127.84, 127.02, 126.40, 114.18, 114.09, 113.17, 113.04, 80.86, 55.14, 55.12.

### 2.2. Synthesis of Compound **3**

Compound **2** (496.6 mg, 0.8 mmol) was dissolved in dry dichloromethane (15 mL), and then boron tribromide (0.28 mL, 3 mmol) in dichloromethane (5 mL) was added to the solution at 0 °C. The reaction mixture was stirred at room temperature and monitored by TLC. After the reaction was completed, the reaction was quenched by saturated sodium bicarbonate (5 mL). The aqueous layer was extracted with dichloromethane three times, and the combined organic layer was dried over anhydrous Na_2_SO_4_. The crude product was purified on a silica-gel column using hexane/ethyl acetate (2:1) as eluent to give compound **3** as a red solid (431.5 mg, 91%). ^1^H NMR (400 MHz, DMSO) (TMS, ppm) *δ* 9.34 (d, *J* = 9.2 Hz, 2H), 7.84 (d, *J* = 8.5 Hz, 2H), 7.70–7.62 (m, 1H), 7.55 (dt, *J* = 21.7, 7.9 Hz, 8H), 7.15 (t, 2H), 7.10 (d, *J* = 6.9 Hz, 1H), 7.05 (d, *J* = 8.7 Hz, 2H), 6.98 (d, *J* = 7.4 Hz, 2H), 6.80 (d, *J* = 7.0 Hz, 2H), 6.76 (d, *J* = 8.5 Hz, 2H), 6.51 (t, 4H). ^13^C NMR (100 MHz, CDCl_3_) *δ* 174.61, 174.59, 154.37, 154.27, 145.22, 144.89, 143.95, 140.81, 138.51, 136.51, 136.22, 136.13, 134.47, 132.82, 132.79, 132.67, 132.10, 131.41, 131.17, 130.52, 128.88, 127.83, 127.02, 126.39, 126.33, 114.73, 114.60, 114.15, 114.06, 80.79.

### 2.3. Synthesis of Compound **4**

Compound **3** (414 mg, 0.7 mmol), tert-butyl 2-bromoacetate (408 mg, 2.1 mmol), and cesium carbonate (0.65 g, 2 mmol) were dissolved in DMF (15 mL) and then stirred at room temperature for 12 h. After the reaction was completed, the solid was filtered off, and the filtrate was concentrated in vacuo to give a residue, which was proportioned in dichloromethane (15 mL) and water (15 mL). The organic layer was separated, and the aqueous layer was extracted with dichloromethane. The combined organic layer was dried over anhydrous Na_2_SO_4_ and concentrated, and then purified by gel column chromatography (eluent: hexane/ethyl acetate = 5:1) to give product compound **4** as a red solid (413 mg, 72%) ^1^H NMR (400 MHz, CDCl_3_) (TMS, ppm) *δ* 7.69–7.67 (d, 2H), 7.64–7.60 (t, 1H), 7.54–7.47 (m, 6H), 7.41 (d, *J* = 8.3 Hz, 2H), 7.18–7.12 (m, 5H), 7.08–7.06 (m, 2H), 7.00–6.95 (m, 4H), 6.68–6.64 (m, 4H), 4.47 (s, 4H), 1.49 (s, 9H), 1.46 (s, 9H). ^13^C NMR (100 MHz, CDCl_3_) (ppm) *δ* 174.51, 167.97, 156.66, 145.17, 144.79, 143.84, 140.61, 138.90, 136.84, 136.57, 136.15, 134.51, 132.66, 132.10, 131.39, 131.15, 130.53, 128.88, 127.86, 127.02, 126.41, 113.78, 113.76, 82.35, 82.33, 65.66, 65.62, 28.04, 28.00.

### 2.4. Synthesis of TPEDPD

To the solution of compound **4** (328 mg, 0.4 mmol) in dichloromethane (10 mL), trifluoroacetic acid (0.13 mL, 1.6 mmol) was added dropwise at 0 °C. The reaction mixture was stirred at room temperature for 8 h. After the reaction was completed, the resulting mixture was diluted with 20 mL dichloromethane and washed with water. The organic phase was separated and dried by Na_2_SO_4_. Evaporation of the solvent under reduced pressure and further purification were carried out by column chromatography using hexane/ethyl acetate (1:1) as eluent to give the desired product as a red solid (212 mg, yield: 75%). ^1^H NMR (400 MHz, DMSO) (TMS, ppm) *δ* 12.95 (s, 2H), 7.85 (d, *J* = 8.1 Hz, 2H), 7.67 (s, 1H), 7.61–7.51 (m, 8H), 7.19–7.14 (m, 3H), 7.09–7.07 (d, *J* = 8.0 Hz, 2H), 7.02–7.00 (d, *J* = 7.3 Hz, 2H), 6.94–6.87 (m, 4H), 6.72–6.67 (t, *J* = 9.7 Hz, 4H), 4.59 (s, 4H). ^13^C NMR (100 MHz, CDCl_3_) (ppm)δ 174.65, 173.36, 156.89, 156.19, 156.09 145.12, 144.53, 143.62, 140.18, 139.37, 137.30, 137.32 136.75, 136.09, 134.54, 132.77, 132.75, 132.71, 132.07, 132.00, 131.37, 131.19, 130.54, 128.90, 127.92, 127.06, 126.56, 126.48, 114.17, 114.05, 113.94, 113.82, 80.81, 64.73.

### 2.5. Preparation of Drug Loaded Liposome

First, hybrid lipid (12 mg HSPC, 3 mg cholesterol, and 1 mg DSPE-mPEG2000-Mal), AIE PS (1 mg) and PTX (1 mg) were dissolved in 2 mL chloroform. The mixed solution was transferred into a 100 mL round-bottomed flask, followed by rotary vacuum evaporation at 55 °C to form a lipid film. Then, 4 mL of 0.5% sodium chloride solution was added to hydrate the film via vigorous stirring at 50 °C. Next, the as-synthesized liposome was extruded back and forth ten times through polycarbonate membrane with a pore size of 200 nm in a mini extruder to obtain PTX/PS@Lip. For Zn^2+^ loading, 1 mL of ZnCl_2_ (2 mg/mL) aqueous solution was mixed with the above PTX/PS@Lip and stirred overnight. The unencapsulated zinc ion was removed by a centrifugal filter (10 kDa MWCO) to achieve the product PTX/PS-Zn@Lip. Finally, 4 mg of AS1411 Apt was added to PTX/PS-Zn@Lip aqueous solution and stirred for 3 h. The final product PTX/PS-Zn@Lip-Apt was purified by a centrifugal filter (10 kDa MWCO).

### 2.6. Measurement of ROS Generation

ABDA was used as an indicator to measure ROS generation in tested samples. ABDA solution (10 μL, 2.05 mg mL^−1^ in DMSO) was mixed with PTX/PS-Zn@Lip-Apt (1 mL, 10 μg mL^−1^ PS) micelles in aqueous solution. Then, the mixed solution was exposed to white light (80 mW cm^−2^). The absorption decrease of ABDA at 378 nm was recorded to obtain the relative ROS generation ability of PS or PS-loaded liposomes.

### 2.7. Cell Culture

PC3 cells were cultured in complete DMEM cell culture medium supplemented with 10% fetal bovine serum and 1% penicillin-streptomycin (penicillin 10,000 U/mL, streptomycin 10 mg mL^−1^) in an incubator containing 5% CO_2_ at 37 °C.

### 2.8. Cellular Uptake

A confocal laser scanning microscope (CLSM) was employed to examine the cellular uptake efficacy of the liposomes. PC3 cells were planted in a 20 mm glass-bottom Petri dish with a density of 1 × 10^6^ cells per dish overnight. Then the cells were treated with liposome at a concentration of 2 mg mL^−1^ PS with or without light irradiation. After 4 h, cells were washed with PBS three times and imaged on a CLSM (LSM900, ZEISS, Oberkochen, Germany) equipped with a 60× oil-immersion objective. The wavelength of the exciting laser was 405 nm, and the emission was collected from 420 nm to 450 nm.

### 2.9. In Vitro Cytotoxicity

For the cytotoxicity study, PC3 cells were seeded in 96-well plates (8 × 10^3^ cells per well) and incubated overnight. Fresh culture media containing various concentrations of various liposomes were added to the wells and incubated for 4 h. After light irradiation, the cells were cultured for another 8 h. Afterward, fresh culture medium was added to the cell dishes after washing with 1× PBS three times. Finally, the relative cell viabilities were evaluated by CCK8 assay based on the manufacture’s instruction.

### 2.10. PCI Enhanced Cytotoxicity

PC3 cells were seeded in 96-well plates at a density of 8 × 10^3^ cells per well and co-incubated with different samples for 4 h. After being washed with PBS, fresh medium was added to each well and the first light irradiation was performed (L_1_, 5 min, white light, 20 mW cm^−2^). After incubation for another 4 h, the cells were exposed to the second light irradiation (L_2_, 10 min, white light, 80 mW cm^−2^). At 8 h post-irradiation, CCK8 measurement was performed following the manufacture’s instruction.

### 2.11. Biodistribution In Vivo

To establish tumor models, human prostate cancer PC3 cells (1 × 10^7^ cells in 100 μL PBS) were subcutaneously inoculated into the right flank of 6-week-old BALB/c nude mice. Tumor growth was monitored every other day using a digital caliper (calculated as 0.5 × length × width^2^). For the biodistribution test, the PTX/Dye@Lip-Apt and PTX/Dye@Lip were intravenously injected into the tumor-bearing mice at the concentration of 2.0 mg mL^−1^ ICG in PBS. Mice were anesthetized with 2.5% (*v*/*v*) isoflurane in 100% oxygen at a flow rate of 1 L/min at given time points and imaged by an animal imaging system (PerkinElmer, Waltham, MA, USA). Imaging was performed during the maintenance phase with 1.5% isoflurane. For tissue distribution studies, the mice were sacrificed after imaging. Heart, liver, spleen, lungs, kidneys, and tumors were excised and imaged.

### 2.12. In Vivo Antitumor Efficacy

When the tumor volume reached about 200 mm^3^, mice bearing PC3 tumors were randomly divided into seven groups (*n* = 5): (1) PBS group (−); (2) PTX@Lip (without light irradiation); (3) PTX/PS@Lip (without light irradiation); (4) PTX/PS@Lip (LL); (5) PTX/PS-Zn@Lip (LL); (6) PTX/PS-Zn@Lip-Apt group (LL); and (7) PTX/PS-Zn@Lip-Apt group (L_1_L_2_). After 3 h of liposome injection, tumor-bearing mice were exposed to white light irradiation for either continuous 20 min (LL group, 80 mW/cm^2^) or 20 min of dual-stage irradiation (L_1_L_2_ group, 20 mW/cm^2^ for 5 min, 80 mW/cm^2^ for 15 min). The same treatment with laser irradiation was repeated on day 4, day 8, day 12, and day 16. The body weight and tumor volume were monitored every other day. The mice were sacrificed after 20 days of treatment. The tumors were collected for photographs, and the main organs (heart, liver, spleen, lung, and kidney) were collected for H&E staining.

## 3. Results

### 3.1. Synthesis and Characterization of AIE Photosensitizer (TPEDPD)

**Figure 2:**
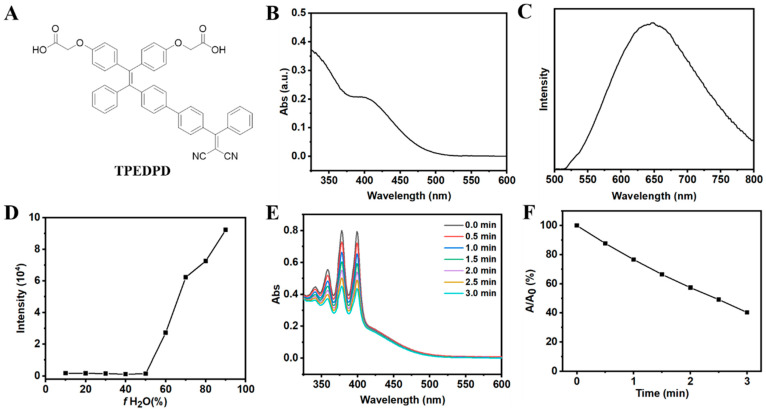
(**A**) Chemical structure of TPEDPD. (**B**) UV-Vis absorption spectra of TPEDPD (10 μg/mL). (**C**) Fluorescence spectra of TPEDPD (10 μg/mL) with excitation at 420 nm. (**D**) Fluorescence intensities of TPEDPD (10 μg/mL) at 650 nm in mixed solvent with various DMSO/H_2_O ratios. (**E**) Changes in the absorption of ABDA induced by ROS generation from TPEDPD (10 μg/mL) in water with white light irradiation (power density: 80 mW cm^−2^). (**F**) Decomposition rates of ABDA induced by ROS generation from TPEDPD (10 μg/mL) in water.

The AIE PS (TPEDPD) was synthesized from compound **1**, as previously described. The synthetic route is presented in Scheme S1. The chemical structure of TPEDPD (Figure 2A) was confirmed through NMR and high-resolution mass spectroscopy (HR-MS) analysis (refer to Figures S1–S9). The absorption and emission spectra of TPEDPD were measured in a dimethyl sulfoxide/water (DMSO/H_2_O) mixture with a ratio of 1/99 *v*/*v*, revealing peaks centered at 420 and 650 nm, respectively (see Figure 2B,C). Subsequently, we characterized the AIE property of TPEDPD in DMSO/H_2_O mixtures with varying ratios. Markedly, the fluorescence intensity of TPEDPD significantly increased with the increase in water ratio, exhibiting a typical AIE-active manner (Figure 2D and Figure S10). Moreover, TPEDPD showed good photosensitizing capability, as confirmed by its ability to decompose 9,10-anthracenediylbis(methylene)dimalonic acid (ABDA) upon white light irradiation (see Figure 2E,F).

### 3.2. Preparation and Characterization of Liposome

**Figure 3:**
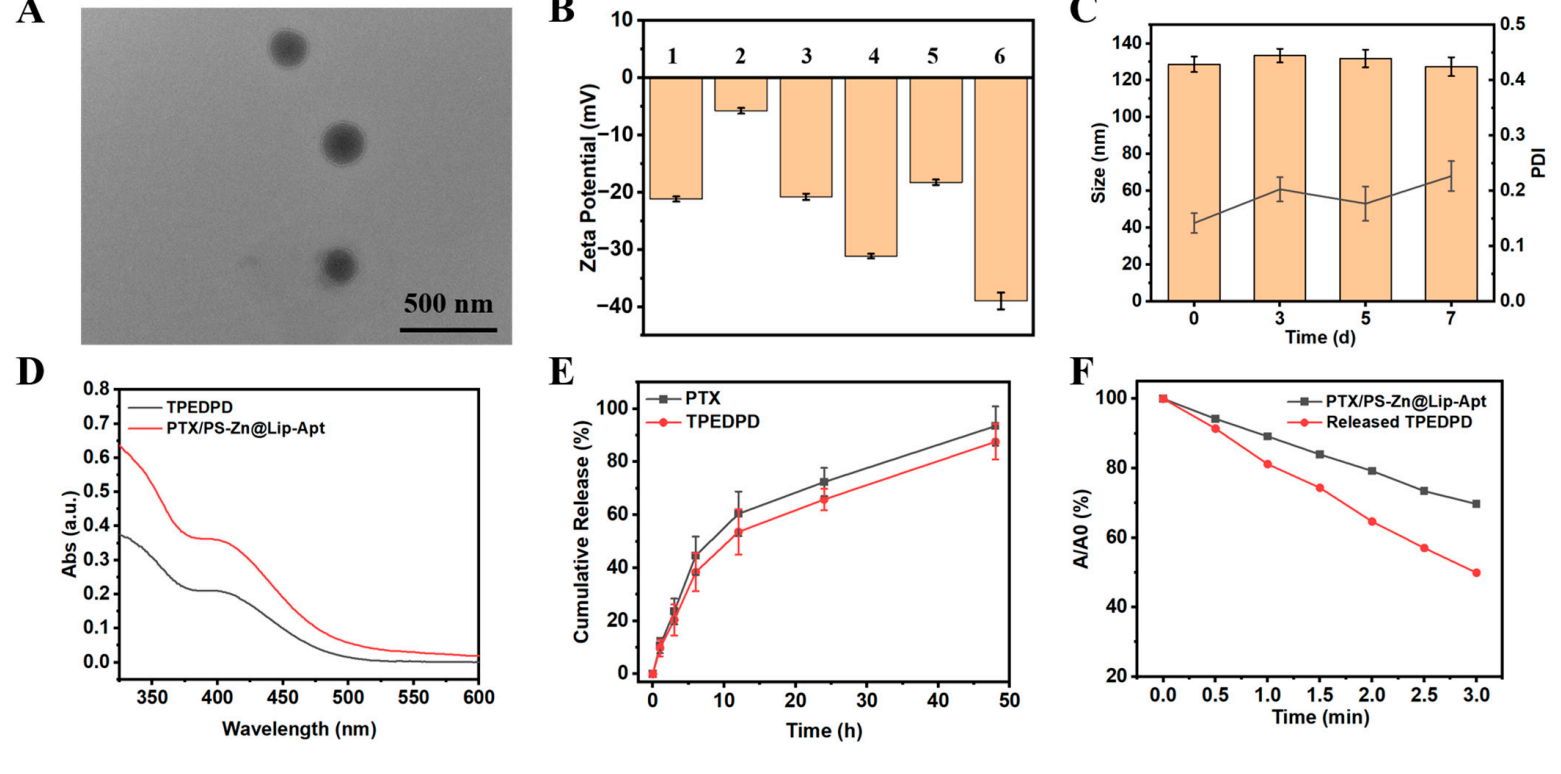
(**A**) TEM image of PTX/PS-Zn@Lip-Apt. (**B**) Zeta potential of various liposomes dispersed in water. 1: Lip, 2: PTX@Lip, 3: PS@Lip, 4: PTX/PS@Lip, 5: PTX/PS-Zn@Lip, and 6: PTX/PS-Zn@Lip-Apt. (**C**) The average diameter and PDI of PTX/PS-Zn@Lip-Apt at various time points, stored in the dark at 4 °C. (**D**) UV-Vis absorption spectra of TPEDPD (10 μg/mL) and PTX/PS-Zn@Lip-Apt (10 μg/mL based on TPEDPD). (**E**) Release of PTX and TPEDPD from PTX/PS-Zn@Lip-Apt in PBS (0.1 M, pH = 7.4) with 0.1% Tween 80 at 37 °C. (**F**) Decomposition rates of ABDA induced by ROS generation from TPEDPD (10 μg/mL) in water before and after release from PTX/PS-Zn@Lip-Apt.

In this versatile liposome, PTX and PS were ensconced within the hydrophobic layer, and Zn^2+^ was loaded by chelation with the carboxyl group of TPEDPD. As shown in Figure S11, the bare liposome and PTX@Lip solution was milky, while the solutions of AIE PS-loaded liposomes were yellow and opaque. The prepared liposomes showed a spherical morphology (Figure 3A and Figure S12), with an average size ranging from 120 to 180 nm (Table S1). Furthermore, as shown in Figure 3B, all liposomes displayed a negative surface charge. These liposomes also demonstrated high drug-loading capabilities for both PTX and TPEDPD, achieving encapsulation efficiencies (EEs) exceeding 88% (Table S1). Notably, PTX/PS-Zn@Lip-Apt maintained its stability without any observable precipitation or aggregation when stored at 4 °C for a duration of 7 days. There were no discernible changes in its average diameter or zeta potential during this storage period (see Figure 3C and Figure S13). Furthermore, as displayed in Figure S14, less than 2.5% of PTX and TPEDPD leaked from PTX/PS-Zn@Lip-Apt during this time frame, underscoring the remarkable stability of PTX/PS-Zn@Lip-Apt under these conditions.

The absorption and emission of PTX/PS-Zn@Lip-Apt were assessed using ultraviolet–visible (UV/vis) absorption and fluorescence spectroscopy. In comparison to free TPEDPD, there is a noticeable enhancement in both the absorption and fluorescence intensities of PTX/PS-Zn@Lip-Apt (Figure 3D and Figure S15). Such enhancement may be ascribed to the AIE effect of TPEDPD, as the phospholipids of the liposome layer suppressed the intramolecular motions and nonradiative decay of TPEDPD. Next, the real-time release of PTX and TPEDPD from PTX/PS-Zn@Lip-Apt was monitored (Figure 3E). The cumulative release of PTX and TPEDPD from PTX/PS-Zn@Lip-Apt was 93.5% and 87.6%, respectively, after 48 h, demonstrating a sustained release effect. The release performance of Zn^2+^ was also evaluated using ICP-MS, which displayed a similar drug release kinetic to PTX and TPEDPD (Figure S16). Then, the capacity of PTX/PS-Zn@Lip-Apt to induce ROS generation under laser irradiation was assessed using a singlet oxygen probe (^1^O_2_), ABDA. As shown in Figure 3F and Figure S17, interestingly, it was found that the ^1^O_2_ generation ability of TPEDPD in PTX/PS-Zn@Lip-Apt was quenched to a certain extent. The reason for this phenomenon is elusive, and we will further investigate it in our future work. After being released from the liposomes, the ^1^O_2_ generation ability of TPEDPD recovered. Such a property of this system can reduce the phototoxicity of the photosensitizer before it accumulates in the tumor sites and is released from the liposomes.

### 3.3. Cellular Uptake of PTX/PS-Zn@Lip-Apt NPs

**Figure 4:**
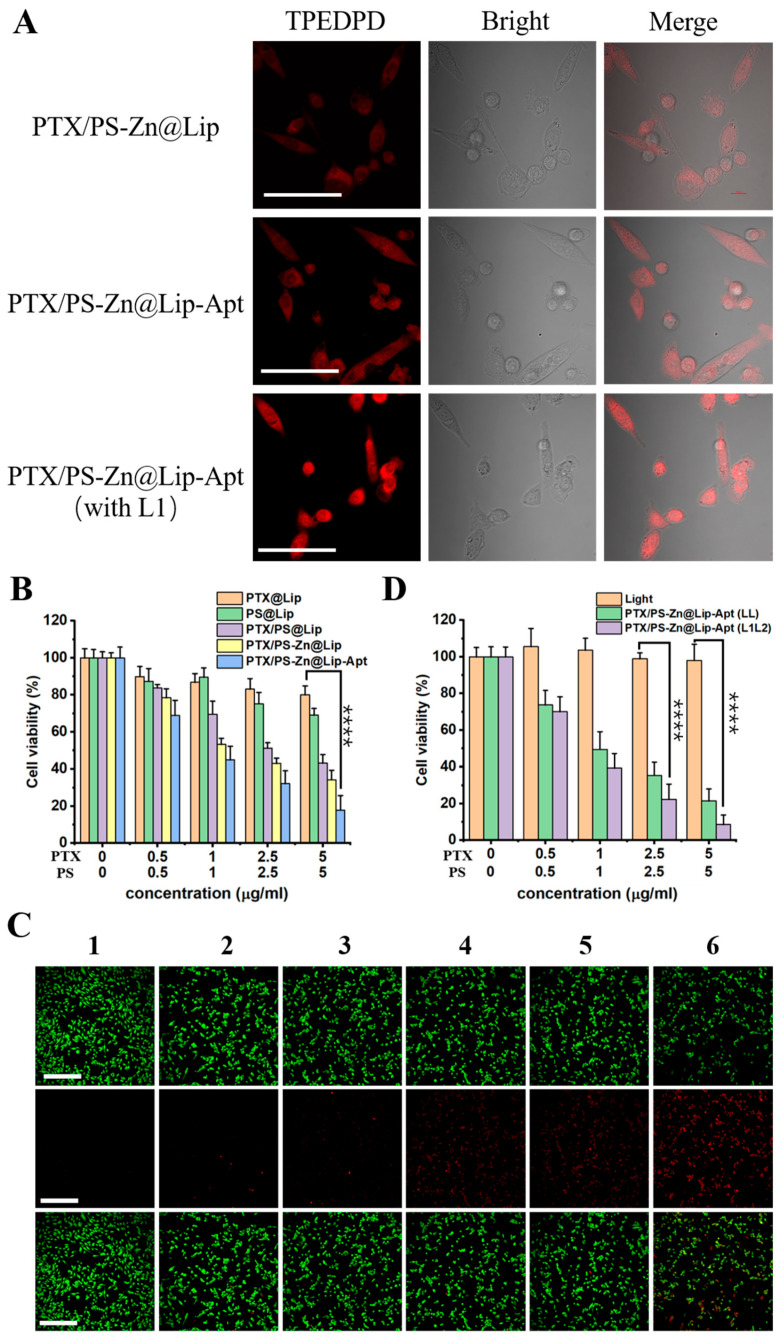
(**A**) CLSM images of PC3 cells after incubation with PTX/PS-Zn@Lip and PTX/PS-Zn@Lip-Apt with or without the first light irradiation (L1). The scale bar is 100 μm. (**B**) PC3 cell viability study after incubation with different formulations. (**C**) Live/dead cell staining analysis with different treatments. (1: PBS, 2: PTX@Lip, 3: PS@Lip, 4: PTX/PS@Lip, 5: PTX/PS-Zn@Lip, and 6: PTX/PS-Zn@Lip-Apt). The scale bar is 100 μm. (**D**) The influence of the PCI effect on PC3 viability study. Statistical analysis was performed by two-way ANOVA (*n* = 3, **** *p* < 0.0001).

To demonstrate the enhanced cellular uptake by PCI effect, the cellular internalization of PTX/PS-Zn@Lip and PTX/PS-Zn@Lip-Apt NPs was studied by CLSM. The distribution of the liposome was determined based on the inherent fluorescence of the AIE PS. As shown in Figure 4A, the red fluorescence was mainly detected in the cytoplasm of PC3 cancer cells after an incubation time of 3 h. Compared with the weak intracellular fluorescence of the PTX/PS-Zn@Lip group, Apt-targeted groups exhibited much stronger fluorescence, indicating far more internalization of liposome. Thus, incorporating AS1411 Apt onto liposomes can enhance the cellular uptake for NCL-positive cells. Furthermore, when PC3 cells were exposed to the first light irradiation (L_1_, 5 min), the observed red fluorescent intensity was stronger than that of cells without L_1_ light irradiation for PTX/PS-Zn@Lip-Apt NPs. Such a result demonstrated that cellular uptake of the liposome can be enhanced by the PCI effect.

### 3.4. In Vitro Therapeutic Effect

We subsequently investigated the in vitro therapeutic effect. CCK-8 assay was employed to evaluate the cytotoxicity of different liposomes. As exhibited in Figure 4B, compared with liposomes loaded with a single chemotherapeutic drug or photosensitizer, the photoinduced cytotoxicity of PTX/PS@Lip was significantly higher, which was ascribed to the synergistic effect of combined chemo/photodynamic therapy. For Zn^2+^-loaded liposomes, the tumor cell killing effect was more pronounced. When incubated with PTX/PS-Zn@Lip-Apt under continuous laser irradiation, the cell viability decreased significantly to around 18% due to the targeting ability of Apt. As shown in Figure 4C, live/dead cell staining analysis indicated a similar result with CCK-8 assay. To test whether the PCI effect can improve the tumor suppression efficacy, a dual-stage light irradiation was conducted. A first light irradiation (L_1_, 5 min) was performed during cellular internalization, followed by a second-time light irradiation (L_2_, 10 min) after cellular internalization (Figure 4D). First, negligible cytotoxicity was detected for the control group with only light irradiation. Compared with cells treated with PTX/PS-Zn@Lip-Apt (LL), the cytotoxicity was further enhanced when treated with PTX/PS-Zn@Lip-Apt (L_1_L_2_), demonstrating that the PCI effect of PS can enhance the therapeutic effect of NPs due to the improved cell internalization. Furthermore, the cytotoxicity difference of PTX/PS-Zn@Lip-Apt on the normal cell line and tumor cell line was evaluated (Figure S18). It was found that PTX/PS-Zn@Lip-Apt showed a higher toxicity to PC3 cells than to NIH3T3 cells. This may be because the AS1411 aptamer can specifically bind to nucleolin, which is overexpressed in prostate cancer.

### 3.5. In Vivo Fluorescence Imaging

**Figure 5:**
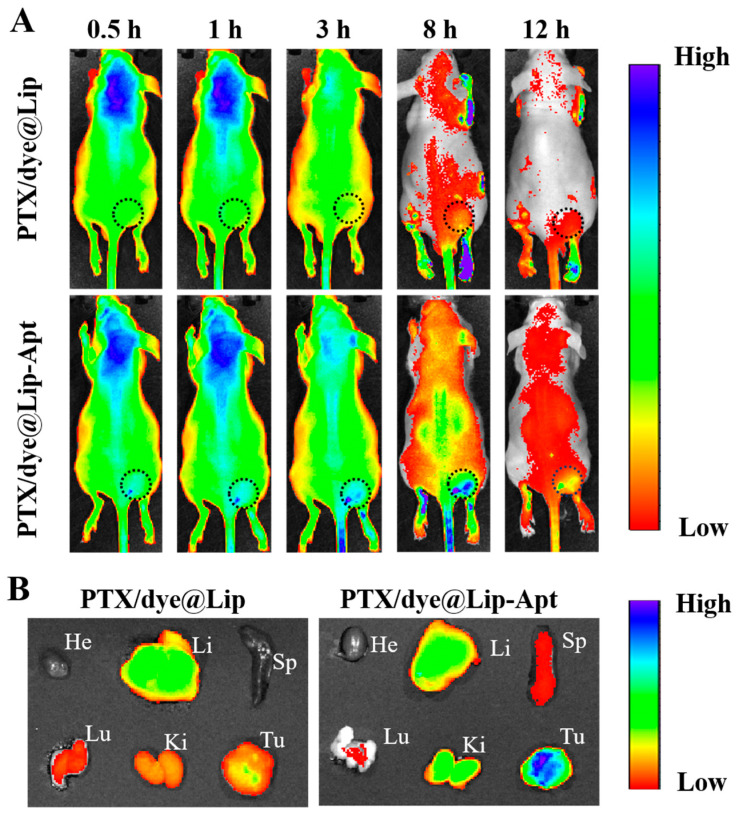
(**A**) In vivo fluorescence images of PC3 breast-tumor-bearing mice after i.v. injection of different NPs (100 μL of 2 mg mL^−1^ based on ICG). The black dashed circle indicates the location of the tumors. (**B**) Ex vivo imaging of tumors and major organs at 8 h post-injection (He: heart, Li: liver, Sp: spleen, Lu: lung, Ki: kidney, Tu: tumor).

The in vivo biodistribution of the liposome was evaluated in PC3 tumor-bearing mice by using a IVIS Lumina XRMS Series III system (PerkinElmer, Waltham, MA, USA). As shown in Figure 5A, the fluorescence intensity of PTX/Dye@Lip-Apt gradually increased at the tumor site and reached a maximum within 8 h. By contrast, PTX/Dye@Lip accumulated in tumor tissue at only 3 h post-injection and then underwent rapid clearance. The fluorescence intensity of both PTX/Dye@Lip and PTX/Dye@Lip-Apt in the tumor sites declined over time because of drug metabolism, but the fluorescence of PTX/Dye@Lip-Apt was still observed 12 h after injection. The fluorescence signal of PTX/Dye@Lip-Apt in the tumor is much higher than that of PTX/Dye@Lip, which resulted from the excellent tumor-targeting ability of Apt conjugation. Figure 5B shows the ex vivo fluorescence imaging of resected main organs and tumors. For PTX/Dye@Lip, the NPs mainly accumulated in the liver. By contrast, PTX/Dye@Lip-Apt-treated mice had the highest fluorescence in the tumor site, confirming again the outstanding tumor-targeting ability of Apt.

### 3.6. In Vivo Enhanced Anticancer Effect Based on the Combined Treatments

**Figure 6:**
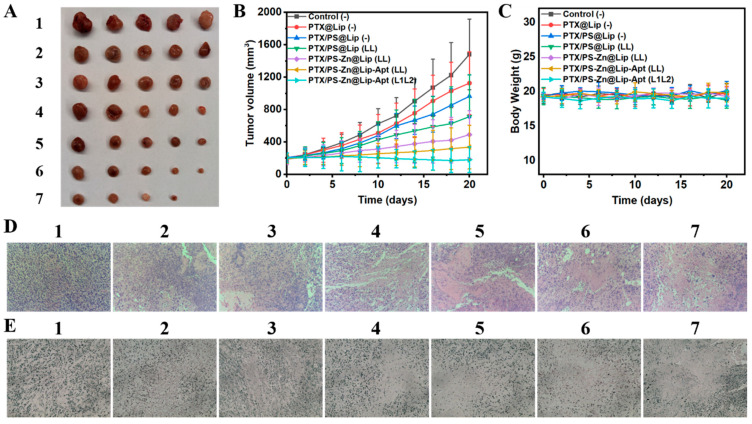
(**A**) Tumor images of the different groups after 20 days intravenous injection treatment. (**B**) Tumor volume change after PBS and various NPs (10 mg kg^−1^ based on TPEDPD, 100 μL) treatment (LL: samples received continuous irradiation; L1L2: samples received dual-stage light irradiation). (**C**) Body weight change analysis of tumor-bearing mice. (**D**) H&E analysis of tumor sections. (**E**) Histological immunofluorescence in tumor sites labeled with TUNEL. 1: PBS, 2: Lip, 3: PTX@Lip, 4: PS@Lip, 5: PTX/PS@Lip, 6: PTX/PS-Zn@Lip, and 7: PTX/PS-Zn@Lip-Apt. The microscope’s magnification in (**D**,**E**) is 20×.

Encouraged by the excellent cellular killing ability of PTX/PS-Zn@Lip-Apt in vitro, we then assessed its in vivo anticancer efficacy in PC3 tumor-bearing mice. As presented in Figure 6B, after 20 days of treatment, the tumor volume of all treated groups was smaller than that in the PBS group. Injection of PTX/PS@Lip without white light irradiation showed a similar tumor growth inhibition rate to that in the PTX@Lip group. However, once subjected to certain light irradiation, the growth of the tumor was further inhibited in the PTX/PS@Lip (LL) group. Compared with the PTX/PS@Lip (LL) group, tumor growth was significantly retarded after intravenous injection of Zn^2+^-loaded liposomes with light irradiation. The tumor inhibition rate of mice treated with PTX/PS-Zn@Lip-Apt (LL) was higher than that of mice treated with PTX/PS-Zn@Lip (LL), implying the good antitumor effect of zinc ions and the excellent tumor-targeting ability of Apt. Impressively, the tumor size in the PTX/PS-Zn@Lip-Apt (L_1_L_2_) treatment group was significantly smaller than that in all other groups. These results were also verified by the representative tumor images (Figure 6A), suggesting that the strategy of dual-stage light irradiation could improve the effect of zinc ion-enhanced chemo-photodynamic therapy. The change in body weight of mice was used to evaluate the systemic toxicity. As shown in Figure 6C, the body weight was stable during the treatment, indicating the weak systemic toxicity of drug-loaded liposomes.

H&E histological staining and histological immunofluorescence showed that tumors in the PTX/PS-Zn@Lip-Apt (L_1_L_2_) treatment group had the most severe architectural destruction of tumor tissue (Figure 6D,E). The treatment with PTX/PS-Zn@Lip-Apt (L_1_L_2_) showed minimal toxicity, as evidenced by no detectable histopathological changes in major organs compared with the PBS group (Figure S19).

## 4. Discussion

The effectiveness of a single treatment method, such as chemotherapy or radiotherapy, for prostate cancer is limited. In this study, we developed an AIE PS, zinc ion, and PTX co-loaded liposome for zinc-enhanced chemo-photodynamic combined therapy for prostate cancer. Additionally, AS1411 aptamer was modified onto the surface of liposome and the PCI strategy was also applied.

Compared to existing chemo-photodynamic systems for prostate cancer treatment, we introduced zinc ions into our nanoplatform. Zinc ions have been shown to inhibit the growth of prostate cancer and chemosensitivity to some chemotherapeutic drugs can also be enhanced when administered simultaneously with zinc ions for prostate cancer cells. The aggregation-induced emission photosensitizer TPEDPD avoids the quenching effect of conventional PSs, thus improving its fluorescence in its aggregation state. The introduction of the AS1411 aptamer mediates tumor-selective uptake, while the application of PCI enhances endosomal escape efficiency. This dual-targeting strategy improves the active targeting and cellular uptake ability of such therapeutic agents.

The PS TPEDPD showed a characteristic AIE property in a solvent mixture of DMSO/H_2_O. The good photosensitizing capability of TPEDPD was confirmed using ABDA as a probe. The morphology of PTX/PS-Zn@Lip-Apt was examined using TEM. The prepared liposomes showed a spherical morphology with an average size ranging from 120 to 180 nm. There were no significant changes in the average diameter or zeta potential of PTX/PS-Zn@Lip-Apt during a storage period of 7 days, demonstrating its high stability in water. The encapsulation efficiencies of PTX and TPEDPD both exceed 88%, enabling effective delivery of the therapeutic cargos. Apart from preparative characterization, optical and chemical characterization of PTX/PS-Zn@Lip-Apt was also conducted.

In vitro experiments indicated that aptamer conjugation and PCI application enhanced the cellular uptake and cytotoxicity of PTX/PS-Zn@Lip-Apt. The zinc ion enhanced cytotoxicity could also be found. The in vivo biodistribution of the liposome was evaluated in PC3 tumor-bearing mice by using a small-animal imaging system. The fluorescence signal of Apt-modified liposomes in the tumor was much higher than that of liposomes without Apt, indicating the excellent tumor-targeting ability of Apt. The in vivo experiments demonstrated the good antitumor effect and biosafety of PTX/PS-Zn@Lip-Apt. Overall, these results provide a theoretical and important basis for innovatively applying zinc-enhanced combined chemo-photodynamic therapy in prostate cancer.

In the future, we will focus on the application of our system in immunotherapy. Both PDT and PTX may induce immunogenic cell death (ICD), while zinc ions could trigger the cGAS-STING pathway activation. Our therapeutic platform has inherent potential for synergistic integration with cancer immunotherapy.

## 5. Conclusions

In summary, we presented a tumor-targeting liposome of PTX/PS-Zn@Lip-Apt consisting of an AIE photosensitizer TPEDPD, a chemotherapeutic agent PTX, and zinc ions to enhance combined chemo-photodynamic therapy using the dual-stage light irradiation strategy. It was demonstrated that the combination of chemotherapy and PDT showed an enhanced therapeutic effect. The introduction of zinc ions further heightened the treatment efficiency of the combined therapy against prostate cancer. The nanoplatform showed good targeting ability due to the conjugation of Apt. Additionally, the PCI effect could enhance the cellular internalization of the NPs.

## Data Availability

Data are contained within the article or Supplementary Materials.

## Supplementary Materials

The following supporting information can be downloaded at: https://www.mdpi.com/article/10.3390/pharmaceutics17040448/s1, Table S1: Characterizations of bare and drug-loaded liposomes; Scheme S1: Synthetic route to AIE photosensitizer TPEDPD; Figure S1: ^1^H NMR spectrum of compound **2** in CDCl_3_; Figure S2: ^13^C NMR spectrum of compound **2** in CDCl_3_; Figure S3: ^1^H NMR spectrum of compound **3** in DMSO; Figure S4: ^13^C NMR spectrum of compound **3** in CDCl_3_; Figure S5: ^1^H NMR spectrum of compound **4** in CDCl_3_; Figure S6: ^13^C NMR spectrum of compound **4** in CDCl_3_; Figure S7: ^1^H NMR spectrum of compound TPEDPD in DMSO; Figure S8: ^13^C NMR spectrum of TPEDPD in CDCl_3_; Figure S9: Mass spectrum of TPEDPD; Figure S10: Fluorescence spectra of TPEDPD (10 μg/mL) in mixed solvent with various DMSO/H_2_O ratios; Figure S11: Various liposomes dispersed in 0.5% NaCl solution; Figure S12: TEM images of (A) Liposome and (B) PTX@Lip; Figure S13: The change in zeta potential of PTX/PS-Zn@Lip-Apt as a function of time stored in the dark at 4 °C; Figure S14: The leakage of TPEDPD and PTX from PTX/PS-Zn@Lip as a function of time stored in dark at 4 °C; Figure S15: Fluorescence spectra of TPEDPD (10 μg/mL) and PTX/PS-Zn@Lip-Apt (10 μg/mL based on TPEDPD) with excitation at 420 nm; Figure S16: Release of Zn^2+^ from PTX/PS-Zn@Lip-Apt in PBS (0.1 M, pH = 7.4) with 0.1% Tween 80 at 37 °C; Figure S17: Changes in the absorption of ABDA induced by ROS generation from (A) PTX/PS-Zn@Lip-Apt (10 μg/mL based on TPEDPD) and (B) released TPEDPD from PTX/PS-Zn@Lip-Apt (10 μg/mL); Figure S18: Cytotoxicity of PTX/PS-Zn@Lip-Apt (10 μg/mL based on TPEDPD) on PC3 cells and NIH3T3 cells; Figure S19. Microscopic images of H&E-stained sections of the major organs after tumor-bearing nude mice were treated with PBS, and PTX/PS-Zn@Lip-Apt (with L1L2).

## Abbreviations

The following abbreviations are used in this manuscript:

PCa	Prostate cancer
PTX	Paclitaxel
PCI	Photochemical internalization
PTT	Photothermal therapy
CDT	Chemodynamic therapy
PDT	Photodynamic therapy
ROS	Reactive oxygen species
Lip	Liposome
Zn	Zinc
ACQ	Aggregation-caused quenching
AIE	Aggregation-induced emission
AIEgens	AIE luminogens
RIM	Restriction of intramolecular motion
EPR	Enhanced permeability and retention
Apt	Aptamer
NCL	Nucleolin
DCM	Dichloromethane
TMS	Tetramethylsilane
DMSO	Dimethyl sulfoxide
DMF	Dimethylformamide
NMR	Nuclear magnetic resonance
PS	Photosensitizer
ABDA	9,10-Anthracenediyl-bis(methylene)dimalonic acid
CLSM	Confocal laser scanning microscope
PBS	Phosphate buffer saline
PC3	Human prostate cancer cells
CCK8	Cell Counting Kit-8
HR-MS	High-resolution mass spectroscopy
EEs	Encapsulation efficiencies
UV	Ultraviolet
vis	Visible
TEM	Transmission electron microscope
H&E	Hematoxylin and eosin
TUNEL	TdT-mediated dUTP Nick-End Labeling
TPEDPD	2,2′-(((2-(4′-(2,2-dicyano-1-phenylvinyl)-[1,1′-biphenyl]-4-yl)-2-phenylethene-1,1-diyl)bis(4,1-phenylene))bis(oxy))diacetic acid
