# Supplementary material for: Prostate Cancer-Targeting Liposome Loaded with Zinc Ion-Coordinated Photosensitizer for Enhanced Chemo-Photodynamic Therapy
Source: Pharmaceutics. 2025 Mar 31;17(4):448. doi: 10.3390/pharmaceutics17040448 (PMC12030104; doi:10.3390/pharmaceutics17040448)
Supplement: Supplementary file 1 [file pharmaceutics-17-00448-s001.zip › pharmaceutics-3502807-supplementary.pdf]

Prostate Cancer-Targeting Liposome Loaded with Zinc Ion-Coordinated Photosensitizer for Enhanced Chemo-Photodynamic Therapy

Li Gao [1,2,†], Zhisheng Tang [3,†], Dongming Xiao [2], Xu Chen [2,*] and Yizhun Zhu [1,*]

1 State Key Laboratory of Quality Research in Chinese Medicine and School of Pharmacy, Macau University of Science and Technology, Macau 999078, China
2 College of Pharmacy, Guilin Medical University, Guilin 541004, China
3 The Second Affiliated Hospital, Guilin Medical University, Guilin 541199, China
* Correspondence: chenxu@glmc.edu.cn (X.C.); yzzhu@must.edu.mo (Y.Z.)
† These authors contributed equally to this work.

Keywords: prostate cancer; zinc ion; photodynamic therapy; tumor targeting; photochemical internalization

Table S1. Characterizations of bare and drug-loaded liposomes.

Liposomes	Diameter[a] (nm)	PDI[b]	EE of PTX (%)	EE of PS (%)
Lip	173 ± 5.39	0.19 ± 0.0078	NA	NA
PTX@Lip	170.4 ± 0.59	0.16 ± 0.018	92.4 ± 4.6	NA
PS@Lip	180.9 ± 4.57	0.19 ± 0.015	NA	88.3 ± 3.7
PTX/PS@Lip	170.0 ± 8.63	0.16 ± 0.029	94.7 ± 4.1	90.5 ± 2.9
PTX/PS-Zn@Lip	144.5 ± 1.92	0.12 ± 0.024	NA	NA
PTX/PS-Zn@Lip-Apt	124.1 ± 5.38	0.18 ± 0.027	90.6 ± 2.7	92.2 ± 1.5

[a] Intensity distribution determined by DLS, [b] PDI- polydispersity index.

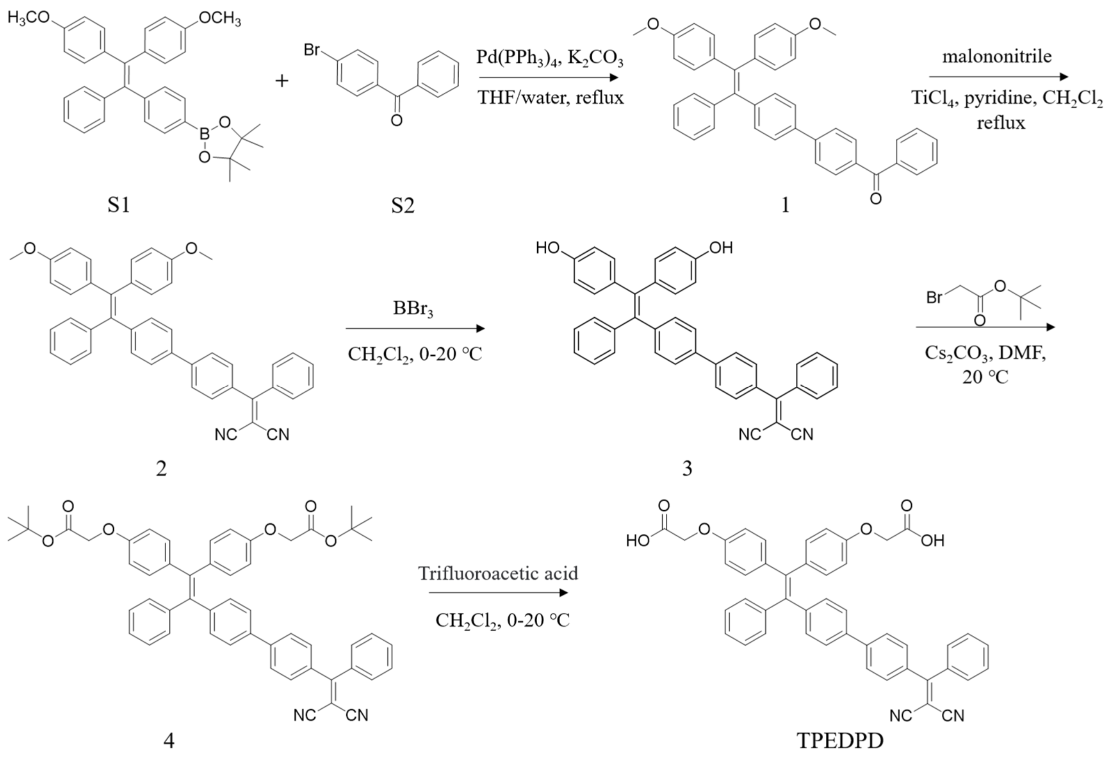

Scheme S1. Synthetic route to AIE photosensitizer TPEDPD.

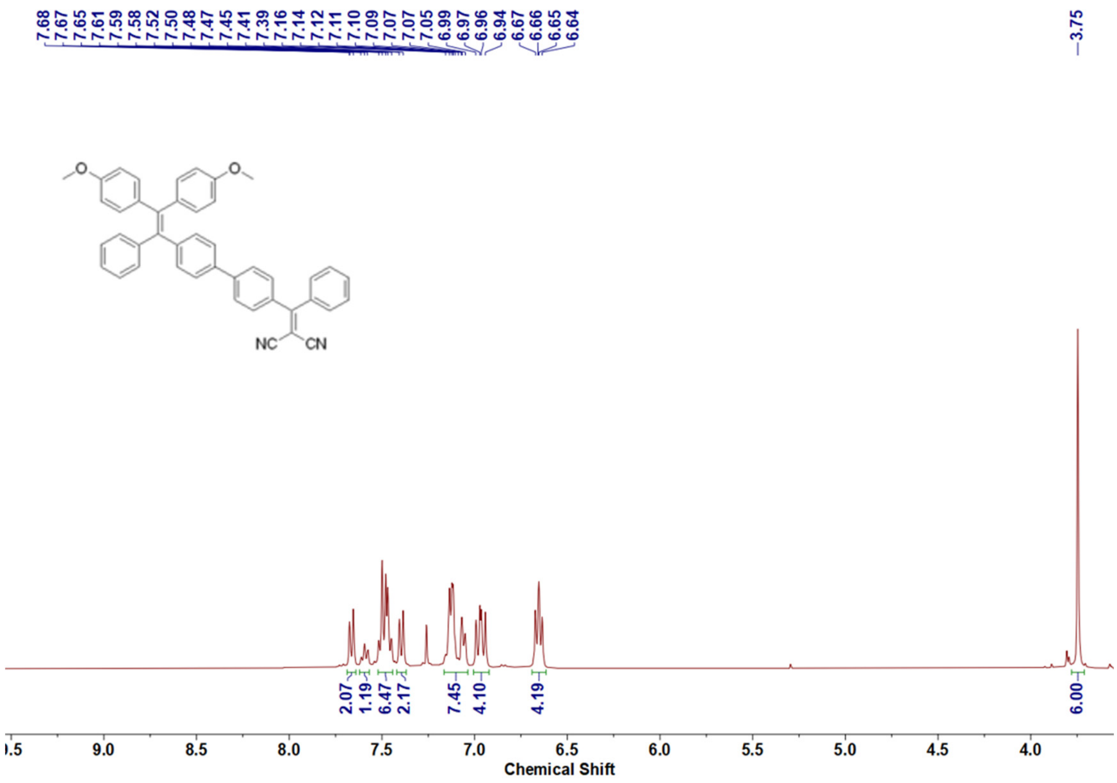

Figure S1. ^{1}H NMR spectrum of compound 2 in $CDCl_3$

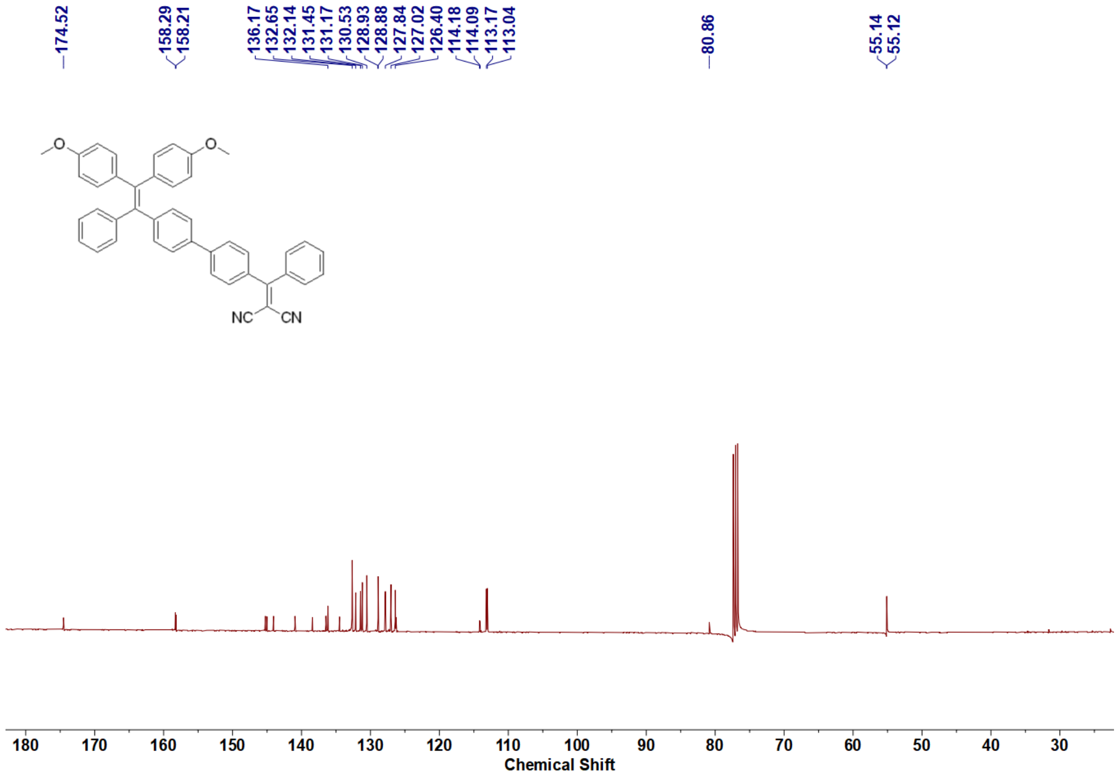

Figure S2. ^{13}C NMR spectrum of compound 2 in $CDCl_3$

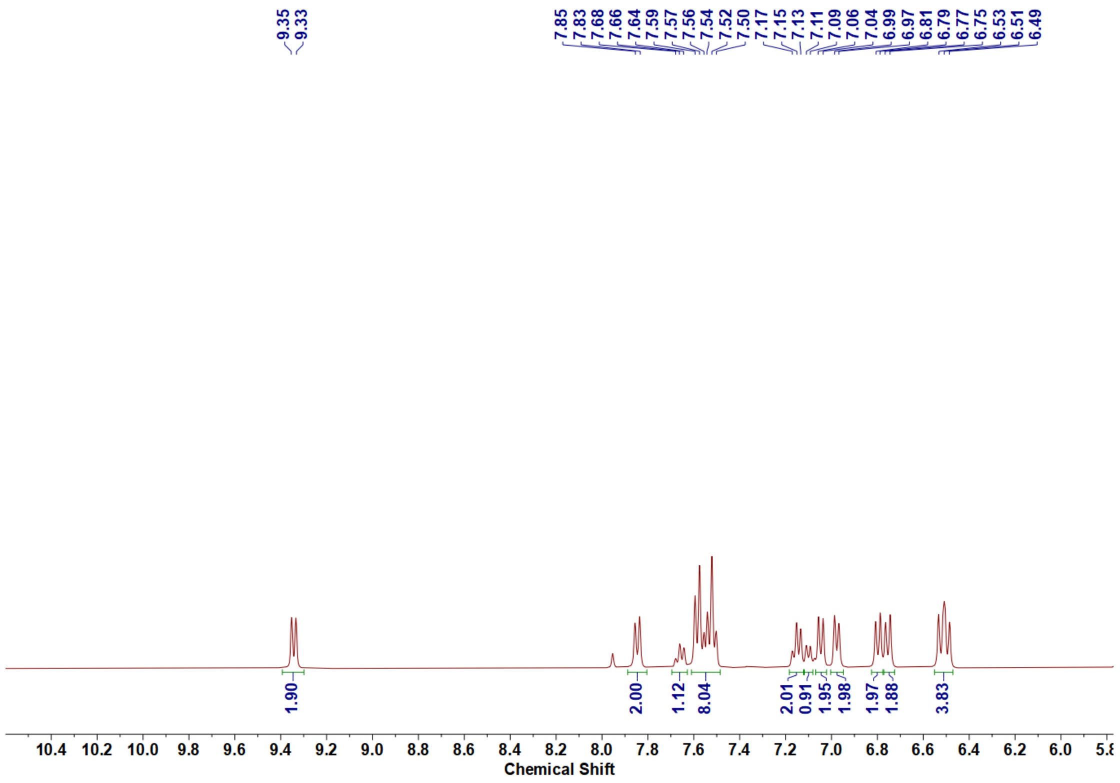

Figure S3. ^{1}H NMR spectrum of compound 3 in DMSO

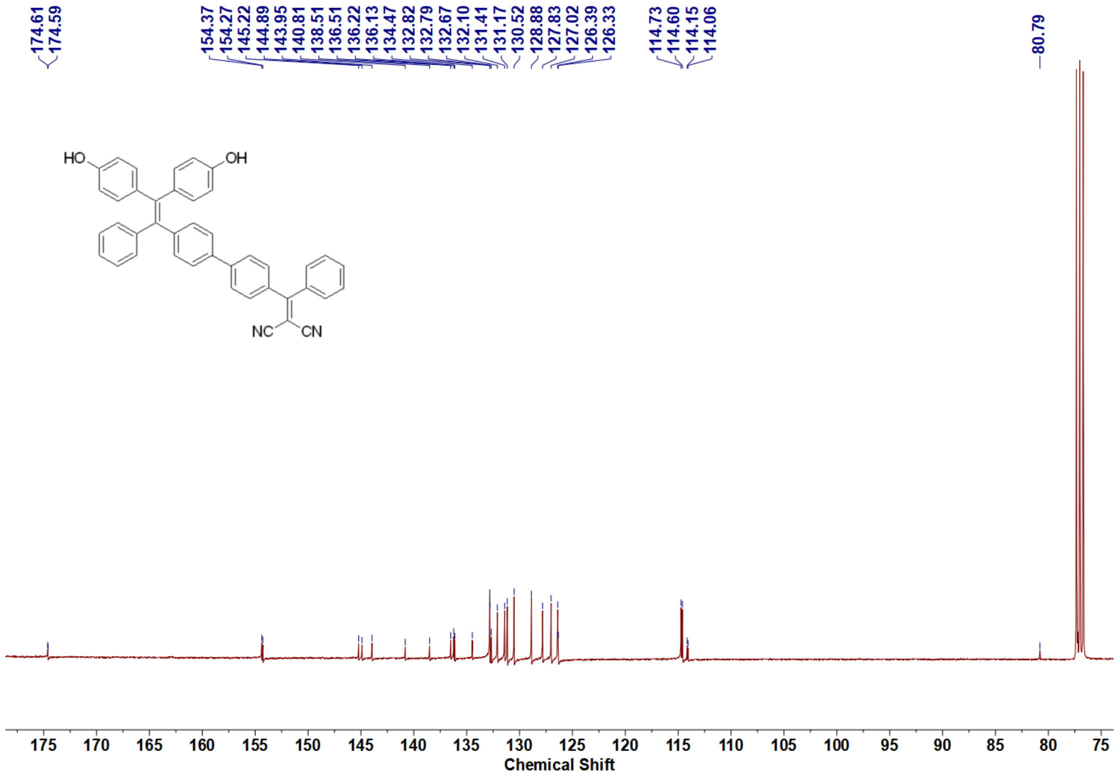

Figure S4. ^{13}C NMR spectrum of compound 3 in $CDCl_3$

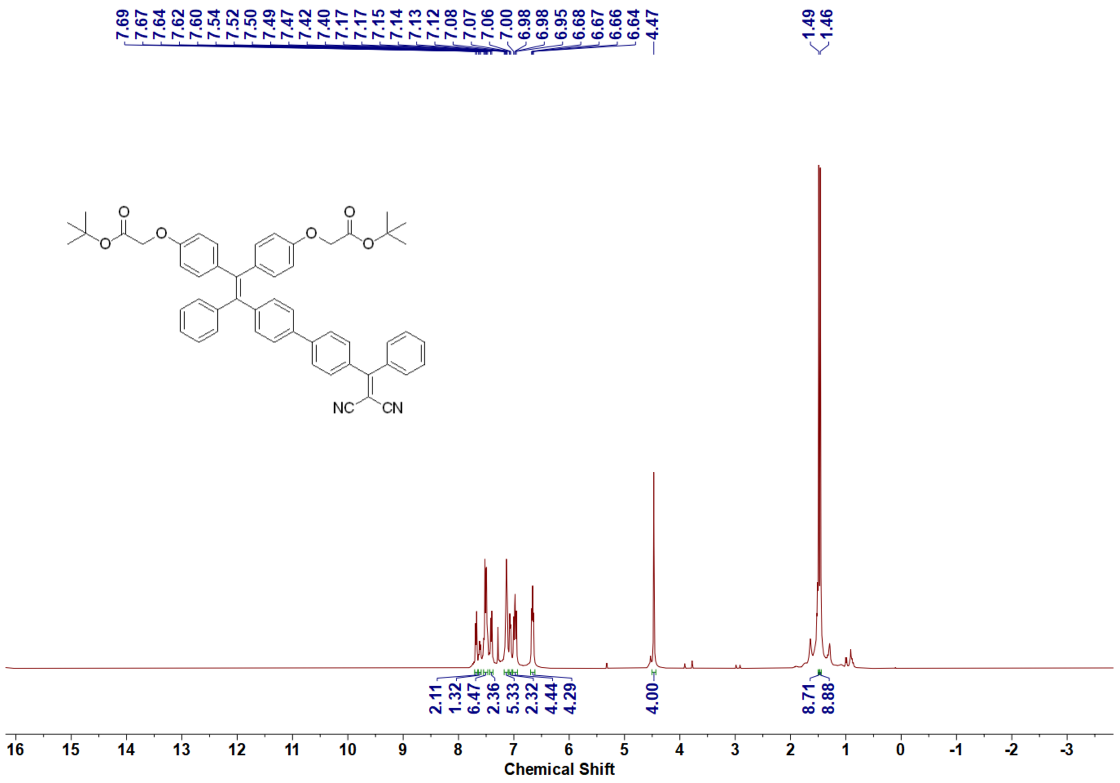

Figure S5. ^{1}H NMR spectrum of compound 4 in $CDCl_3$

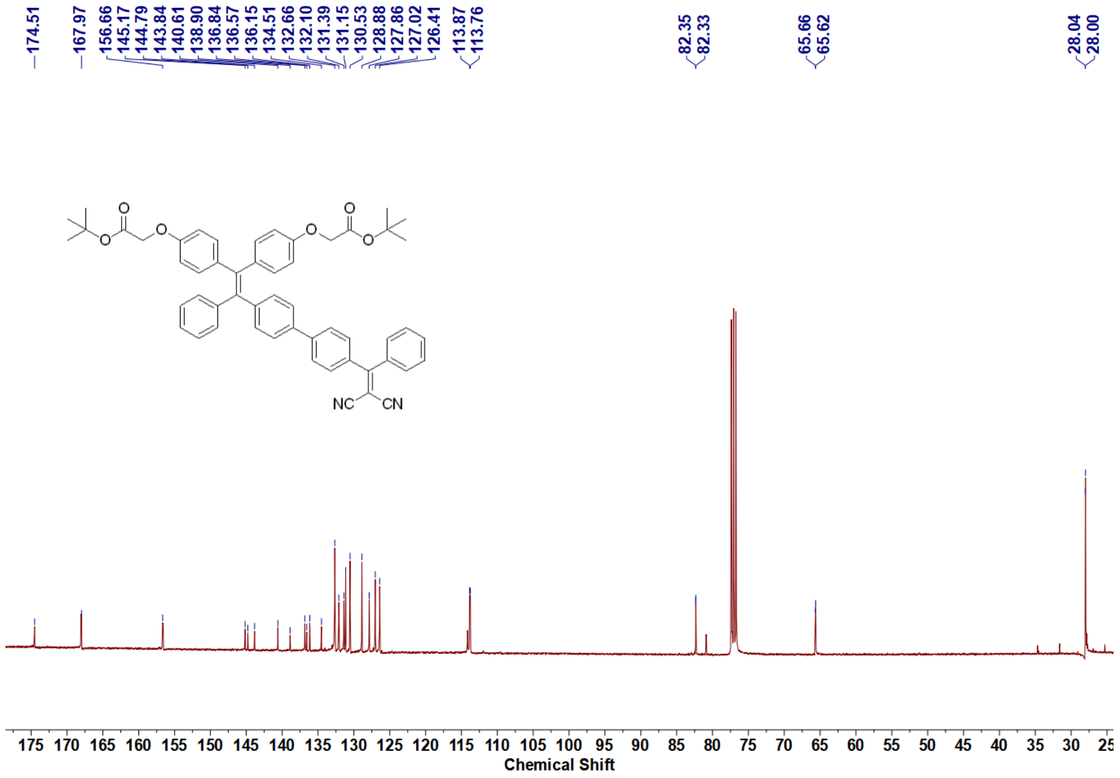

Figure S6. ^{13}C NMR spectrum of compound 4 in $CDCl_3$

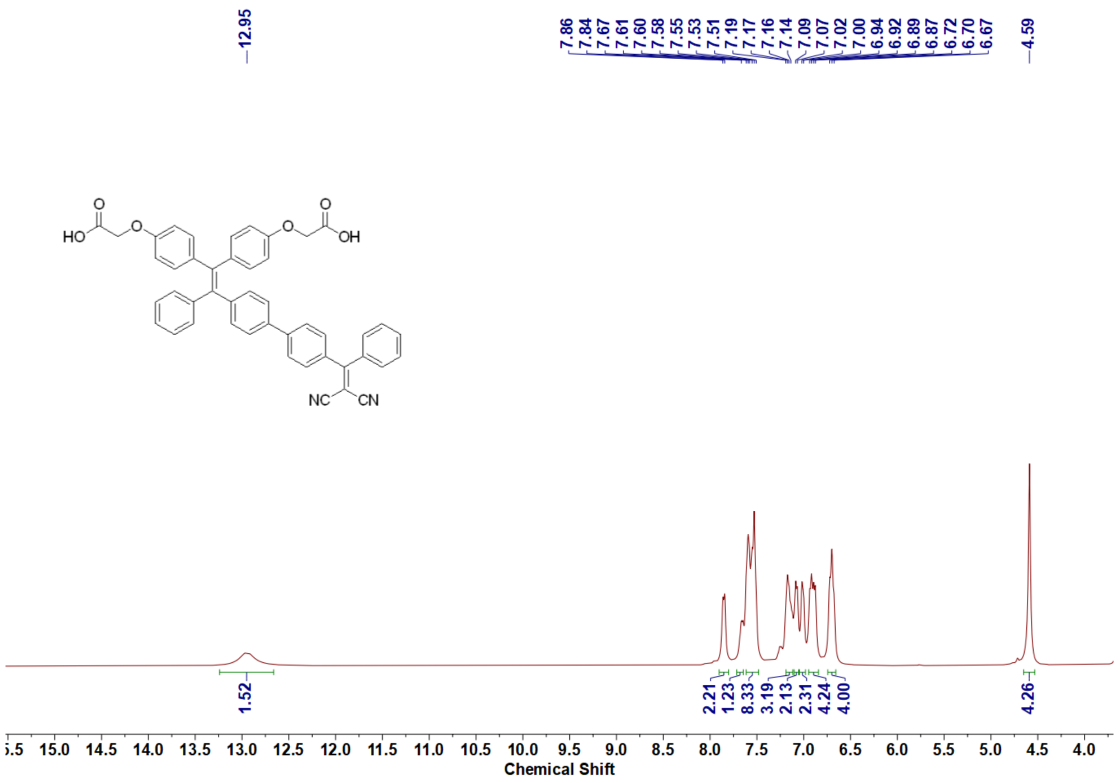

Figure S7. ^{1}H NMR spectrum of compound TPEDPD in DMSO

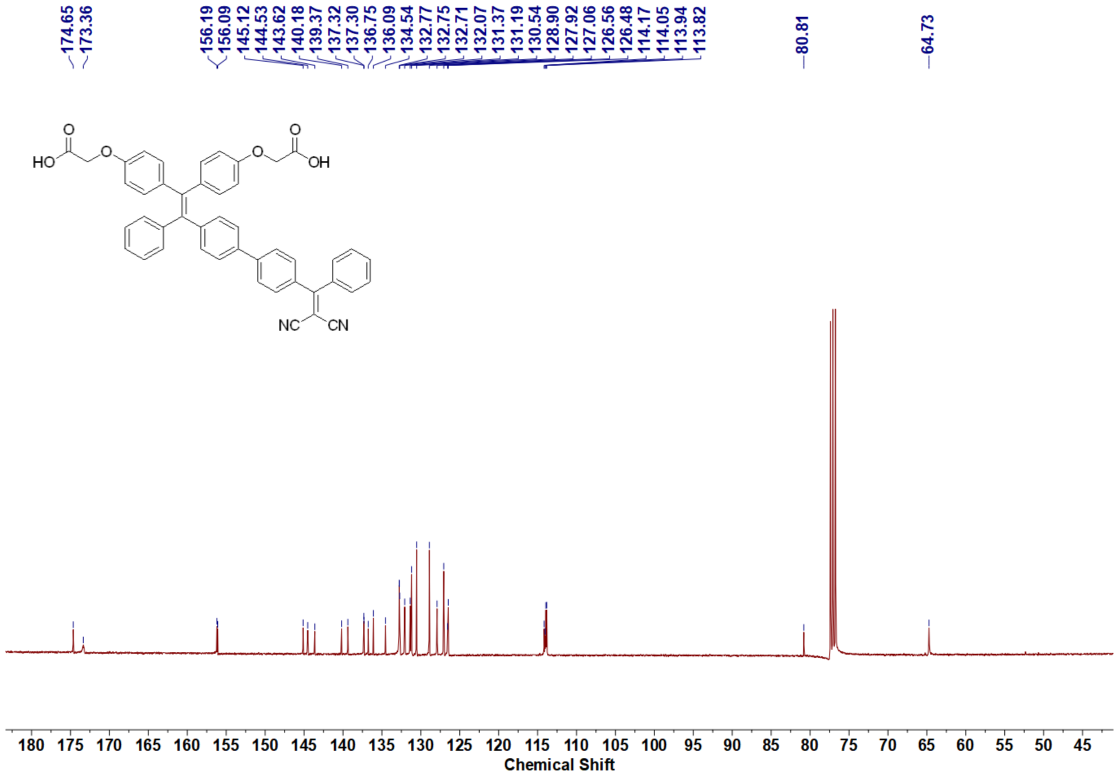

Figure S8. ^{13}C NMR spectrum of TPEDPD in $CDCl_3$

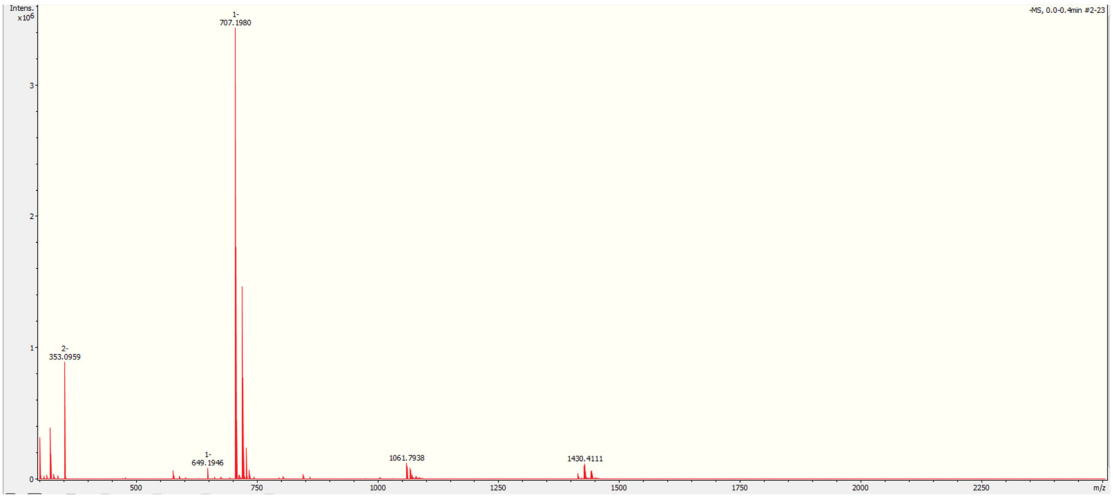

Figure S9. Mass spectrum of TPEDPD

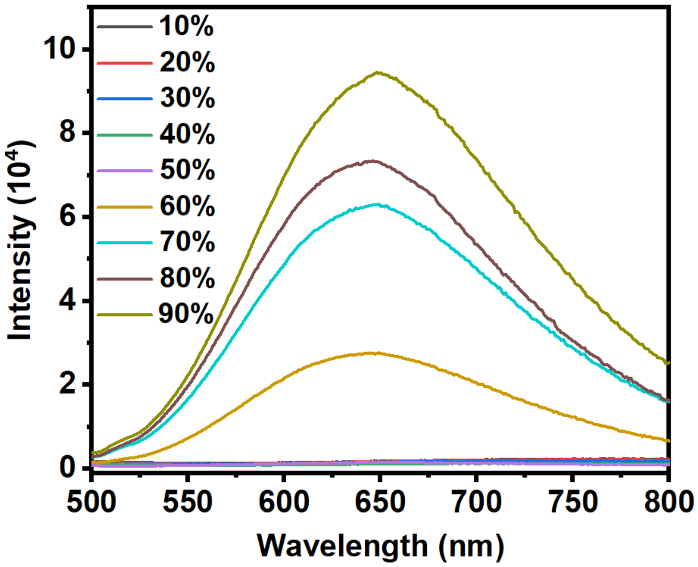

Figure S10. Fluorescence spectra of TPEDPD (10 μg/mL) in mixed solvent with various DMSO/H_2O ratios.

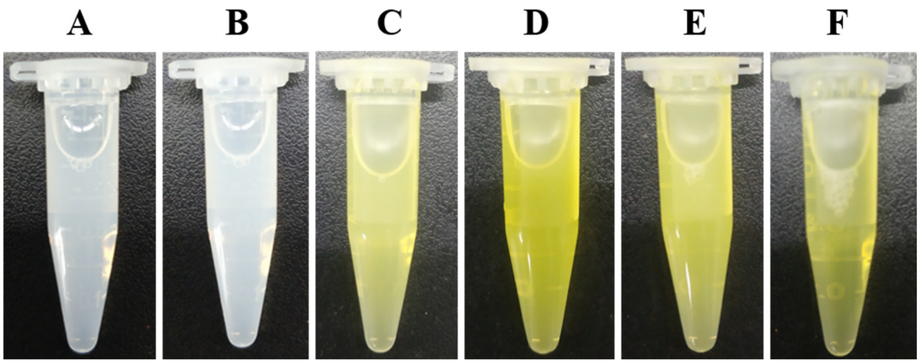

Figure S11. Various liposomes dispersed in 0.5% NaCl solution. (A) Lip, (B) PTX@Lip, (C) PS@Lip, (D) PTX/PS@Lip, (E) PTX/PS-Zn@Lip, and (F) PTX/PS-Zn@Lip-Apt.

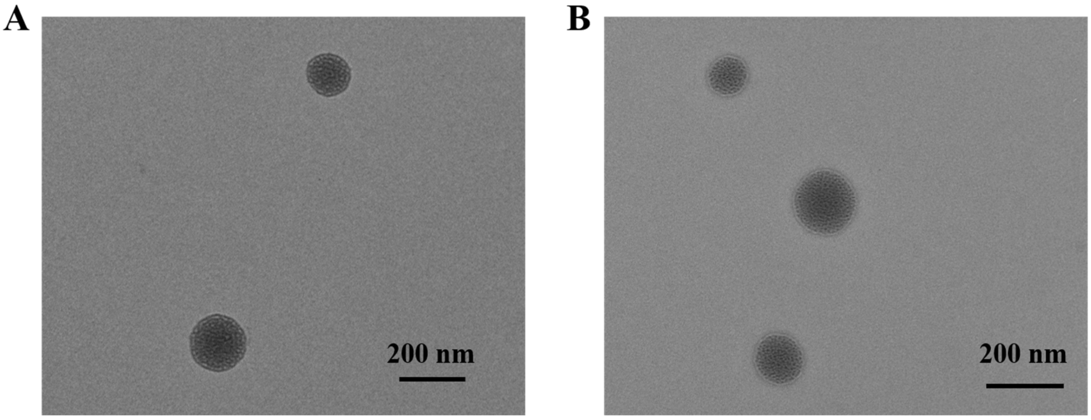

Figure S12. TEM images of (A) Liposome and (B) PTX@Lip.

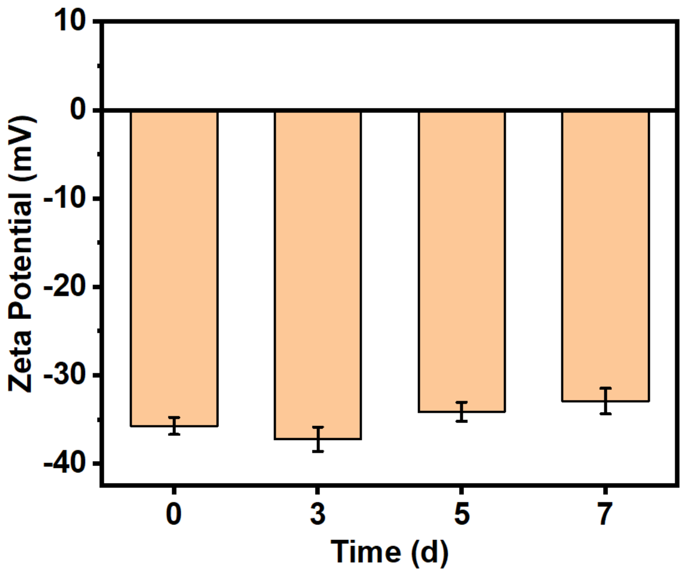

Figure S13. The change of zeta potential of PTX/PS-Zn@Lip-Apt as a function of time stored in dark at 4 ºC.

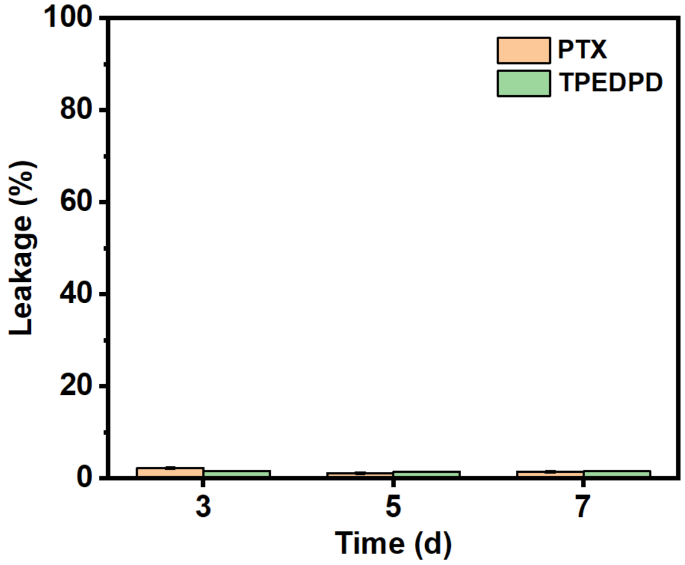

Figure S14. The leakage of TPEDPD and PTX from PTX/PS-Zn@Lip as a function of time stored in dark at 4 ºC.

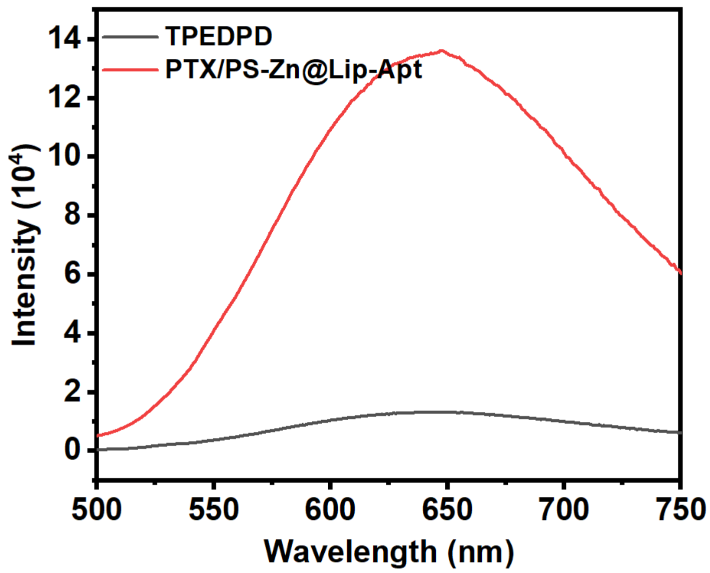

Figure S15. Fluorescence spectra of TPEDPD (10 μg/mL) and PTX/PS-Zn@Lip-Apt (10 μg/mL based on TPEDPD) with excitation of 420 nm.

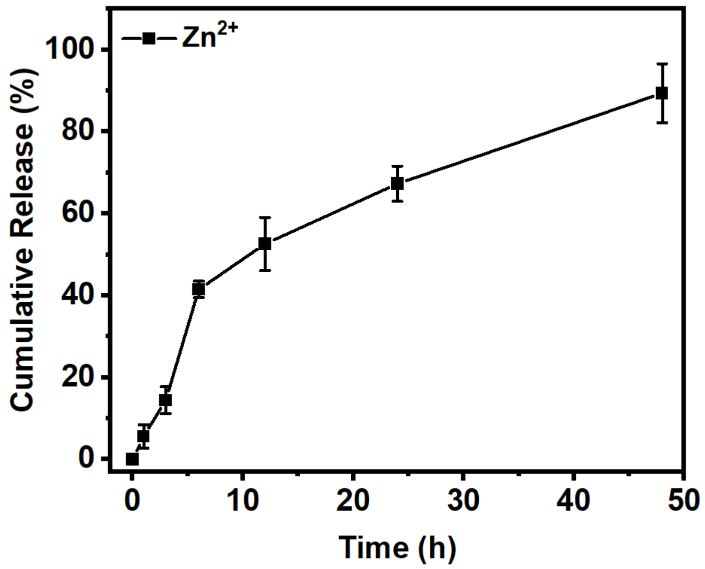

Figure S16. Release of Zn^{2+} from PTX/PS-Zn@Lip-Apt in PBS (0.1 M, pH = 7.4) with 0.1% Tween 80 at 37 ºC.

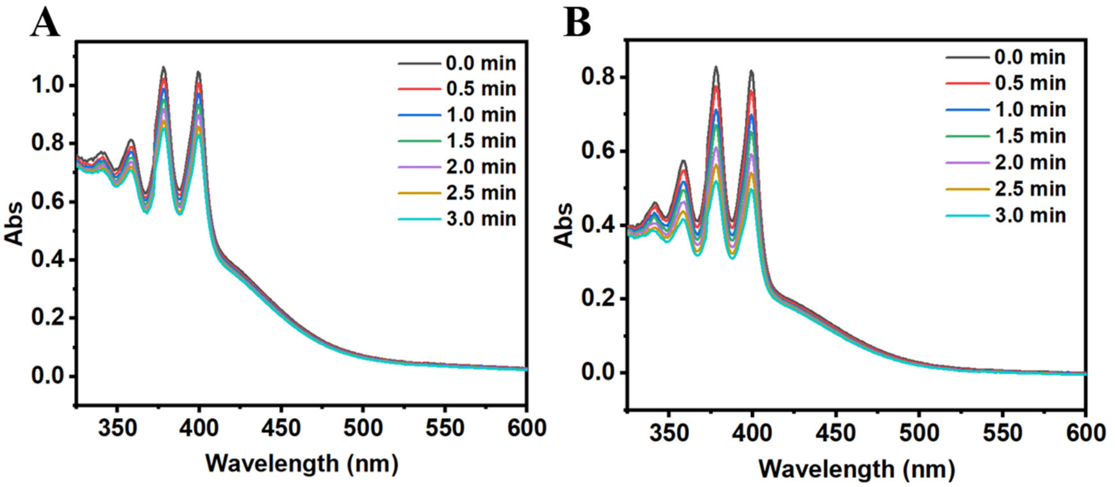

Figure S17. Changes in the absorption of ABDA induced by ROS generation from (A) PTX/PS-Zn@Lip-Apt (10 μg/mL based on TPEDPD) and (B) released TPEDPD from PTX/PS-Zn@Lip-Apt (10 μg/mL).

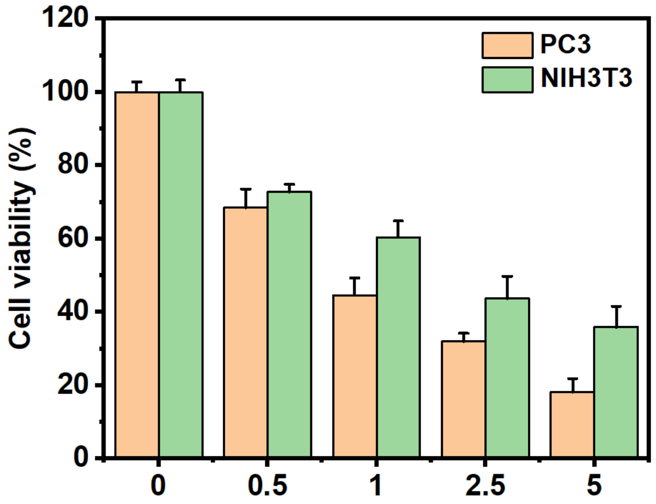

Figure S18. Cytotoxicity of PTX/PS-Zn@Lip-Apt (10 μg/mL based on TPEDPD) on PC3 cells and NIH3T3 cells.

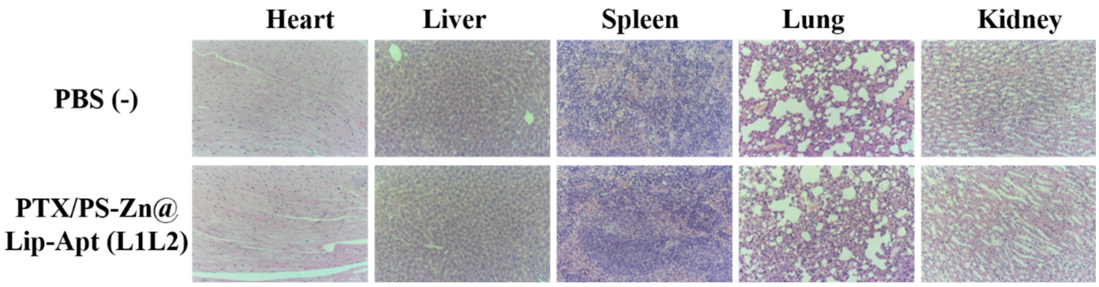

Figure S19. Microscopic images of H&E-stained sections of the major organs after tumor-bearing nude mice being treated with PBS, and PTX/PS-Zn@Lip-Apt (with L1L2).
